# Invasive alien plants in Polish national parks—threats to species diversity

**DOI:** 10.7717/peerj.8034

**Published:** 2019-12-13

**Authors:** Anna Bomanowska, Wojciech Adamowski, Izabella Kirpluk, Anna Otręba, Agnieszka Rewicz

**Affiliations:** 1 University of Lodz, Department of Geobotany and Plant Ecology, Lodz, Poland; 2 Białowieża Geobotanical Station, University of Warsaw, Białowieża, Poland; 3 Botanic Garden, Faculty of Biology, University of Warsaw, Warsaw, Poland; 4 Kampinos National Park, Izabelin, Poland

**Keywords:** Protected areas, Alien flora, Vascular plants, Poland, Biological invasions, Conservation, Invasive species

## Abstract

Due to the relevance of protected areas to the conservation of native biota, the magnitude of invasions and threats posed by alien plants are currently important issues for the preservation of these areas. The paper summarises data on invasive alien plant species presence in the most valuable protected areas in Poland, i.e. national parks (NPs). We investigated the distribution of invasive alien plant species and management attempts concerning those species. We analysed data obtained from 23 national parks originating from published and unpublished sources. Invasive plants were present in all protected areas analysed, from two to 42 species in a particular national park, and 68 in total. The most widely distributed species were: *Impatiens parviflora* (present in 19 NPs), *I. glandulifera* (17), *Solidago gigantea* (17), *Reynoutria japonica* (17), and *Robinia pseudoacacia* (16). The conducted analyses showed that the number of invasive species decreased with the higher altitude (asl) of the national park. The most often managed species were *Impatiens glandulifera* (being removed in seven NPs), *I. parviflora* (six), *Padus serotina* (four) and *Quercus rubra* (four). In the majority of NPs, control activities are limited to small areas and singular species, thus having an incidental character. Only in five objects (Białowieża NP, Biebrza NP, Kampinos NP, Tuchola NP, Wigry NP), management has been focused on several species. We conclude that a lack of comprehensive management of invasive plant species in the majority of national parks currently limits the effectiveness of IAS (invasive alien species) eradication. Exchange of expertise among protected areas, documenting best practice examples, synthesising lessons learnt in IAS management, as well as the development of minimum standards for invasive plants surveillance and management are pivotal.

## Introduction

Protected areas (PAs) play a key role in biodiversity conservation, preserving landscapes and ecosystems which are particularly valuable for nature conservation (Chape et al., 2005; Tittensor et al., 2014; Braun, Schindler & Essl, 2016). In particular, large PAs with strong conservation regime objectives, such as national parks and biosphere reserves, may provide particularly significant benefits for conservation and are the cornerstone of the global protected area network (European Environment Agency, 2012; Le Saout et al., 2013). Thus, the designation and maintenance of those natural, undisturbed ecosystems are part of the most important conservation strategy worldwide (Barber, Miller & Boness, 2004). Protected areas face numerous pressures, including tourism-related issues, wildfire management, poaching and illegal harvesting of resources (Barber, Miller & Boness, 2004; Rands et al., 2010), and climate change (Hannah, Midgley & Millar, 2002). One of the most serious concerns connected with human activity is the spread of invasive alien species (Olaczek, 1998; Foxcroft et al., 2013; Foxcroft et al., 2017).

Basically, natural ecosystems are more resistant to invasions by alien species than anthropogenically transformed areas, and some studies report that PAs contain fewer invasive species than the surrounding areas (Pyšek, Jarošik & Kučera, 2002; Foxcroft et al., 2011; Jarošík, Pyšek & Kadlec, 2011). A high number of native species in the areas with a high degree of naturalness increases competition against alien species and prevents their spreading in PAs (Byers, 2002). Moreover, the level of invasibility of those areas is connected with their protection by law resulting in low disturbance levels, isolation and, in many cases, an association with higher elevation environments, which increases the strength of the climatic barrier for alien species (Pauchard et al., 2009; Foxcroft et al., 2011; Gonzalez-Moreno et al., 2014). However, PAs exist in a matrix of intensive human usage, and many types of human activity disturb ecological sustainability enabling the penetration of alien species into protected areas (McKinney, 2002; Pyšek, Jarošik & Kučera, 2002; Pauchard & Alaback, 2004; Foxcroft et al., 2011; Foxcroft et al., 2013). While only a subset of alien species becomes invasive (Lockwood et al., 2001; Williamson, 2006), a detrimental impact on the environment is often irreversible, especially while facilitated by other drivers such as climate or land use changes (Walther et al., 2009; Pyšek et al., 2013).

Recently, examples showing that alien species can invade natural areas, even those with negligible anthropogenic disturbances, are more frequently reported. Some alien species are capable of crossing ecological barriers and protection boundaries, reaching high mountains (Pauchard et al., 2009; Seipel et al., 2012; McDougall et al., 2011; Kueffer et al., 2013), isolated islands (Walsh et al., 2008; Baret et al., 2013; Shaw, 2013), and relatively undisturbed polar ecosystems (Rose & Hermanutz, 2004; Olech & Chwedorzewska, 2011; Chown et al., 2012).

Vascular plants are one of the taxonomic groups with the most species having a negative impact on natural areas (Lockwood et al., 2001; Braun, Schindler & Essl, 2016; Foxcroft et al., 2017). Currently, the problem of harmful non-native plants that can change habitats in protected areas by destroying the ecosystem structure or modifying natural disturbance regimes, devastating native species, is a global phenomenon (Foxcroft et al., 2013; De Poorter, 2007; Hulme et al., 2014; Monaco & Genovesi, 2014). There is a still increasing body of information containing numbers of non-native plant species in the flora and addressing the impacts of invasive plants on PAs: in Europe (Pyšek, Jarošik & Kučera, 2003; Kleinbauer et al., 2010; Braun, Schindler & Essl, 2016; Dimitrakopoulos et al., 2017; Lapin et al., 2019), Asia (Wu et al., 2009; Hiremath & Sundaram, 2013; Kudo et al., 2014), North (Harrison, Hohn & Ratay, 2002; Rose & Hermanutz, 2004; Allen, Brown & Stohlgren, 2009; Stohlgren, Loope & Makarick, 2013; Abella et al., 2015) and South America (Pauchard & Alaback, 2004; Pivello et al., 2009; Becerra & Simonetti, 2013), Africa (Goodman, 2003; Foxcroft et al., 2013; McConnachie et al., 2015), Australia (Beaumont et al., 2009; Setterfield et al., 2013), and New Zealand (Timmins & Willlams, 1991; West & Thompson, 2013).

Threats posed by different alien plants in PAs are without doubt being recognised as a relevant issue in ecology and conservation biology (Hobbs & Huenneke, 1992), and extensive evidence of a negative impact of invasive aliens on native species and their ecosystems on a global scale suggests that plant invasions may ultimately decrease the potential of PAs to conserve nature (Barber, Miller & Boness, 2004; Rands et al., 2010). The spreading of alien plant species makes it difficult to achieve the basic objectives of protected areas, i.e., the protection of biodiversity (Pyšek et al., 2013; Braun, Schindler & Essl, 2016). Consequently, managing invasive species is a growing challenge (Hulme et al., 2014; Monaco & Genovesi, 2014; Foxcroft et al., 2017).

The magnitude of invasions confronts protected areas with the challenge of prevention and management (Monaco & Genovesi, 2014; Hulme et al., 2014; Foxcroft et al., 2017). This is especially important for Europe, where most protected areas have a long history of anthropogenic usage, including the introduction of alien species (Pyšek, Jarošik & Kučera, 2003; Genovesi et al., 2015; Braun, Schindler & Essl, 2016; Branquart et al., 2016). According to Monaco & Genovesi (2014), 378 invasive plant species in total were distributed in various types of European PAs, and the share of alien species in some of PAs in Europe reached almost 40% (Pyšek et al., 2013).

The pilot study of invasions of alien species of plants and animals conducted in Polish national and natural landscape parks a few years ago indicates that non-native vascular plants are a serious threat to natural and seminatural plant communities as well as native plant species in PAs (Najberek & Solarz, 2011). According to the aforementioned authors, vascular plants constitute as much as 75% (184 species) of all non-native species occurring in PAs, among them, 24 species are considered as invasive in Poland (Tokarska-Guzik et al., 2012). Recently published papers dealing with the occurrence of invasive plant species in particular Polish national parks (e.g.: Dajdok et al., 2007; Sołtys-Lelek & Barabasz-Krasny, 2010; Bomanowska et al., 2014; Radliński, Tronkowska & Tittenbrun, 2015; Wołkowycki, Kołos & Matowicka, 2016) show that the list of invasive plant species threatening these areas is still growing. However, despite the increasing threat posed by alien plants, the magnitude of invasions has not been assessed yet on a country scale.

This article brings a state of the art in the field of plant invasions in Polish national parks (NPs). We collected and analysed a data set of invasive non-native vascular plant species in all national parks in Poland and presented updated data on the occurrence of invasive alien flora in national parks. The aims of the study were: (i) to identify and characterise invasive plant species currently growing in Polish NPs, (ii) to estimate how many invasive plant species were detected in particular NPs and (iii) what were the most and least frequently recorded species among NPs, (iv) to analyse the degree of similarity of invasive plant species composition among NPs, (v) to analyse which variables are important for determining the number of invasive plants across NPs, and (vi) to collect information which invasive alien plants are managed. Findings have implications for early detection and monitoring as well as assessing invasive alien plants management plans in protected areas by providing information on the spread of IAS in different NPs.

## Material and Methods

### Data sources and species attributes used in the study

The study included the most important protected areas in Poland that have their own management authority, i.e., national parks (NP). National parks are large protected areas (usually >100 km^2^) that focus on the conservation of areas of unique natural, cultural, scientific and educational values (IUCN category II) where all nature and landscape values are protected and that follow a minimum intervention approach, i.e., they require that the core zone (cover >75% of the NP) shall be largely kept free of any human influence (Nature Conservation Act, 2004; IUCN, 2016).

**Figure 1:**
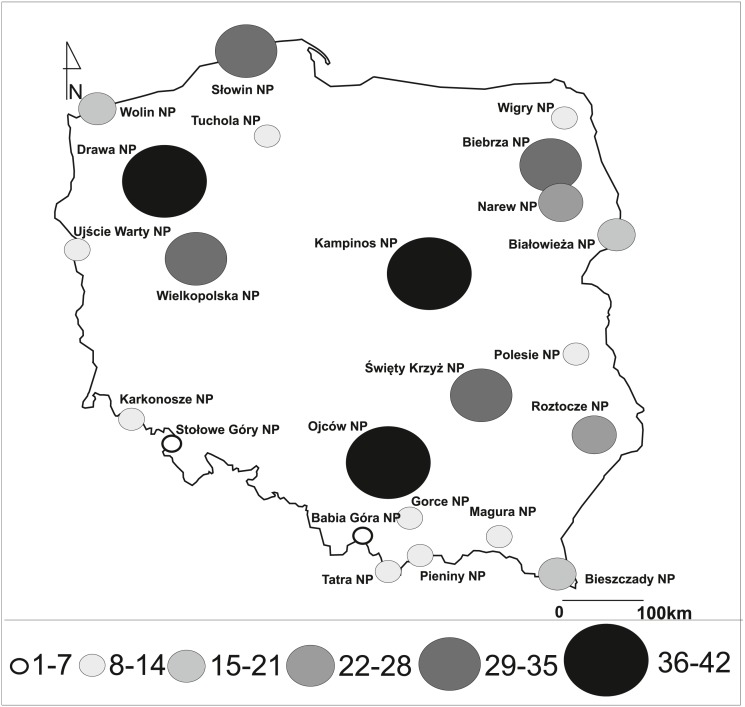
Number of invasive vascular plant species within Polish NPs.

We studied all national parks located in the whole territory of Poland. They differed in their surface area, altitude (asl), and topography, as well as the year of creation, the object of protection, floristic richness and others (Fig. 1, Table 1). In total, 23 NPs were included in the study, of which nine were additionally certified as biosphere reserves, and one was included on the Ramsar List of Wetlands of International Importance (Table 1). Biosphere reserves are also large protected areas focused on the integration of sustainable regional development and conservation where management and development zones are much more extensive than in national parks (UNESCO, 2015). The List of Wetlands of International Importance is defined by the Ramsar Convention for the conservation and sustainable use of wetlands, recognising the fundamental ecological functions of wetlands and their economic, cultural, scientific, and recreational value (Ramsar Convention and United Nations, 1971).

Our research focused exclusively on alien species classified as invasive plants. We used the definition recommended by Richardson et al. (2000) and Pyšek et al. (2004), i.e., invasive plants are naturalised plants that produce reproductive offspring, often in very large numbers, at considerable distances from parent plants, and thus have a potential to spread over a considerable area. In recent years, the threats posed by IAS have become a major topic for consideration by the Convention on Biological Diversity (CBD, 2016) and the International Union for Conservation of Nature (IUCN, 2018) as well as the EU (Genovesi et al., 2015), so we took into account their standpoint and defined invasive alien species as species whose introduction and/or spread threaten biological diversity, species that lead to specific economic losses and species harmful to human and/or animal health. For each protected area, we collected data on the occurrence of invasive alien vascular plants. The complete list of species was prepared based on data excerpted from the literature, including information published in scientific articles and data from the “grey literature”, such as technical reports, lists of species, as well as materials received directly from the staff of national parks (Table 1). We based our work on the most current data, mainly from the 21st century. The oldest data were from the second half of the 20th century (the oldest paper was published in 1979), but they were updated with new information (from 2014–2018) obtained from the staff of national parks (Table 1). We considered only species meeting the following criteria: (i) species listed as invasive plants in Poland at the national, regional or local levels (Tokarska-Guzik et al., 2012); (ii) invasive species in Poland according to the Regulation of the Minister of the Environment of 9th September 2011 on the list of alien plant and animal species whose introduction into the environment may threaten indigenous species or natural habitats (Regulation of the Minister of the Environment, 2011).

**Table 1:** Overview of national parks analyzed in the study.

No	National park	Year of creation	Area (ha)	Elevation range (m)	Total number of vascular plant species	No of invasive vascular plant species	Share of invasive species in the entire flora (%)	Management of invasive plants (no of eradicated species)	Data source
1.	Babia Góra^a^	1954	3391	1025	ca. 650	2	ca. 0.31	1	Babiogórski Park Narodowy, 1979; Kuligowska B. pers. comm.
2.	Białowieża^a^^c^	1947	10517	36	809	20	2.47	3	Adamowski & Keczyński, 1998; Adamowski, Dvorak & Ramanjuk, 2002; Adamowski, 2009; Karczewska M. pers. comm.
3.	Biebrza^b^	1993	59223	27	ca. 900	33	ca. 3.67	9	Tałałaj, Brzosko & Pirożnikow, 2013; Brzosko et al., 2016; Werpachowski & Biereżnoj-Bazille, 2015
4.	Bieszczady^a^	1973	29201	696	ca. 800	16	ca. 2.00	No data	Zemanek & Winnicki, 1999; Kucharzyk S. pers. comm.
5.	Drawa	1990	11342	35	924	42	4.65	No data	Drawieński Park Narodowy, 2018
6.	Gorce	1981	7031	610	ca. 850	20	ca. 2.35	No data	Kozłowska-Kozak & Kozak, 2015; Czarnota P. pers. comm.
7.	Kampinos^a^	1959	38549	35	ca. 1400	40	ca. 2.86	4	Otręba & Ferchmin, 2007; Obidziński, Kołaczkowska & Otręba, 2016; Bomanowska et al., 2014, Authors’ own research
8.	Karkonosze^a^	1959	5581	902	>1000	8	ca. 0.80	5	Fabiszewski & Kwiatkowski, 2001; Fabiszewski & Brej, 2008; Przewoźnik L. pers. comm.
9.	Magura	1995	19439	496	771	10	1.30	1	Perzanowska et al., 2014; Dajdok & Pawlaczyk, 2009; Sochacki J. pers. comm.
10.	Narew	1996	7350	53	ca. 660	28	ca. 4.24	No data	Wołkowycki, Kołos & Matowicka, 2016; Laskowska I. pers. comm.
11.	Ojców	1956	2146	173	ca. 1000	39	ca. 3.90	No data	Barabasz-Krasny, Sołtys & Popek, 2004; Sołtys-Lelek & Barabasz-Krasny, 2010; Sołtys-Lelek A. pers. comm.
12.	Pieniny	1954	2346	532	ca. 1100	7	ca. 0.64	1	Wróbel, 2008; Braun M. pers. data
13.	Polesie^a^	1990	9764	33	ca. 1000	10	ca. 1.00	No data	Poleski Park Narodowy, 2018; Kolasa Sz. pers. comm.
14.	Roztocze	1974	8483	130	ca. 700	27	ca. 4.00	1	Lorens et al., 2013; Radliński, Tronkowska & Tittenbrun, 2015; Radliński B. pers. comm.
15.	Słowin^a^	1967	21573	115	911	33	3.62	No data	Piotrowska, Żukowski & Jackowiak, 1997; Sobisz & Truchan, 2008; Sobisz & Antkowiak, 2009
16.	Stołowe Góry	1993	6340	519	ca. 650	5	ca. 0.77	No data	Fabiszewski & Brej, 2008; Mańkowska-Jurek D. pers. comm.
17.	Święty Krzyż	1950	7626	332	1015	31	3.05	No data	Bróż & Kapuściński, 2000; Sikora & Sobieraj, 2015
18.	Tatra^a^	1954	21197	1599	ca. 1000	11	ca. 1.10	1	Cichocki & Danielewicz, 1993; Dajdok & Pawlaczyk, 2009; Skrzydłowski T. pers. comm.
19.	Tuchola^a^	1996	4613	30	634	11	1.74	5	Park Narodowy Bory Tucholskie, 2010
20.	Ujście Warty	2001	8074	30	ca. 500	8	ca. 1.60	No data	Cieślik Ł. pers. comm.
21.	Wielkopolska	1957	7584	32	ca. 1120	37	ca. 3.30	No data	Żukowski et al., 1995; Danielewicz & Maliński, 1997; Wielkopolski Park Narodowy, 2018
22.	Wigry	1989	15000	57	>1000	13	ca. 1.20	10	Dajdok et al., 2007; Krzysztofiak & Krzysztofiak, 2015
23.	Wolin	1960	8133	116	>900	20	ca. 2.22	1	Woliński Park Narodowy, 2004; Dylawerski M. pers. comm.

**Notes.**

a UNESCO Biosphere Reserve.

b area included on the Ramsar List of Wetlands of International Importance.

c UNESCO World Heritage site.

For each selected invasive plant species, the following information was collected:

• life span –i.e., the morphological type of the plant concerned with its adaptation to ecological conditions: an annual plant, a biennial plant, a perennial plant, a shrub, or a tree. In addition, aquatic plants and climbers are distinguished (Klotz, Kuhn & Durka, 2002);
• area of origin –i.e., the geographical area in which the species occurs naturally, its native range, according to Tokarska-Guzik et al. (2012);
• geographical-historical group –i.e.: archaeophyte –species alien to the natural flora of a given area which arrived and became permanently established before the end of the 15th century (in prehistoric times, in ancient times or during the Middle Ages); kenophyte (=neophyte *sensu* in most Central European studies); species alien to the natural flora of a given area which arrived and became permanently established after the 15th century, starting from the period of great geographical discoveries (conventionally from the date of the discovery of America; Tokarska-Guzik et al., 2012).
• classes of invasiveness of species according to Tokarska-Guzik et al. (2012); i.e.: I –weeds, able to appear in large numbers, mainly in anthropogenic habitats, or potentially invasive species, II; species in which invasive properties are already detected in some regions based on the increasing area of occupancy or the number of localities, or which are invasive in other countries, III; species which occur in Poland in a few localities in large numbers or are scattered over many localities, admittedly in small numbers, but with a known negative impact on native species, habitats and ecosystems and/or on the economy and society, IV; the most dangerous invasive plants, the significance of the presence of those species in Poland is fundamental; both a substantial number of localities and large local populations are known; most are still increasing in terms of number of localities or area of occupancy. The adopted criteria for the identification of invasiveness status of alien species in Poland are in accordance with the guidelines of the European and Mediterranean Plant Protection Organisation (EPPO) for risk assessment of species (Brunel et al., 2010).

Moreover, species considered as the most harmful for European PAs (Monaco & Genovesi, 2014) were indicated.

Data about the number of eradicated invasive plants, which species are managed and which methods are applied were obtained from the literature and technical reports (Table 1). These are estimates, as many NPs lack information on the subject or the information is very general.

Nomenclature of plants follows Mirek et al. (2002).

### Data analysis

For each national park, we calculated the total number of invasive non-native species and the frequency of each species. Occurrence frequency in national parks was used as a measure of species commonness (Brower & Zar, 1984). Due to a somewhat diversified extent of floristic studies carried out at various locations, data on species occurrence were encoded in a binary fashion (0-1, absent-present), without taking into account the degrees of quantitative occurrence. For each species, the frequency of occurrence (Fi) was calculated.

Similarity of invasive alien flora in individual national parks was determined using cluster analysis based on the Euclidean distance (Van Emden, 2008). The Detrended Correspondence Analysis (DCA) was used to assess the variability between national parks. Spearman’s rank correlation coefficients were used to examine relationships between pairs of the following features: the area of national park, altitude, the total number of vascular plant species, and the number of invasive plants.

The software package STATISTICA PL. ver. 10 and Canoco for Windows 4.5 were used for all the above-mentioned numerical analyses (StatSoft Inc., 2013; Lepš & Šmilauer, 2003).

## Results

As a result, 68 taxa of invasive vascular plants which belong to 55 genera and 28 families were found in Polish national parks (Table S1). It is 89.50% of the total number of invasive alien plants in Poland (according to the aforementioned criteria). Over 35% species belong to the families: Asteraceae (15 species; 22.10%) and Poaceae (9; 13.20%). The most numerous group were: perennials (23; 33.80%), annuals (19; 27.90%) and woody species (17; 25.00%).

**Figure 2:**
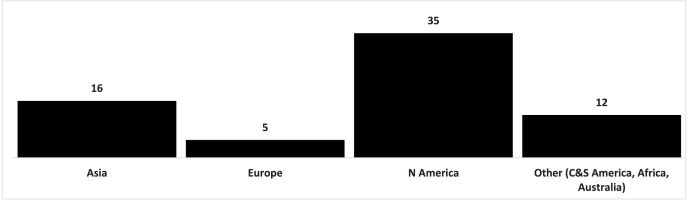
Origin of invasive vascular plant species within Polish NPs.

Foreign invasive plants came mainly from North America (Table S1). There were 35 species from this region, which constituted 51.50% of all invasive plants found in national parks in Poland. There were far fewer species from Asia (16 species; 23.52%) and Europe (5; 7.30%). Species from other regions (Central America and South America, Africa, Australia) had a small share (Fig. 2).

Almost all mentioned species were kenophytes (over 90.00% of all species). Only six were archaeophytes: *Alopecurus myosuroides, Avena fatua, Echinochloa crus-galli, Hordeum murinum, Setaria pumila* and *S. viridis* (Table S1).

**Figure 3:**
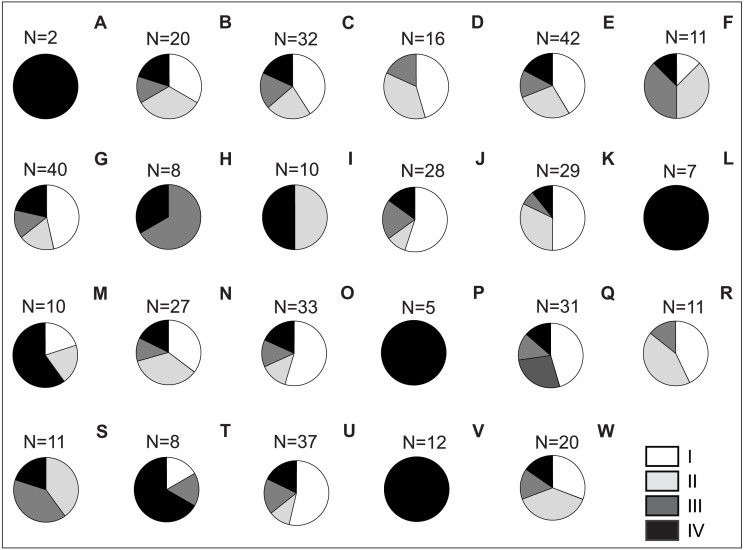
Share of invasive vascular plant species within Polish NPs depending on the category of invasiveness. Explanation: A) Babia Góra NP, B) Białowieża NP, C) Biebrza NP, D) Bieszczady NP, E) Drawa NP, F) Gorce NP, G) Kampinos NP, H) Karkonosze NP, I) Magura NP, J) Narew NP, K) Ojców NP, L) Pieniny NP, M) Polesie NP, N) Roztocze NP, O) Słowin NP, P) Stołowe Góry NP, Q) Święty Krzyż NP, R) Tatra NP, S) Tuchola NP, T) Ujście Warty NP, U) Wielkopolska NP, V) Wigry NP, W) Wolin NP.

Risk assessment of invasive plants in Polish national parks shows that up to 24 species (35.30%) belong to IV category of invasiveness in Poland (according to the categorisation of Tokarska-Guzik et al., 2012; Table S1). The most dangerous invasive plants in this category were recorded in all national parks, and in four parks (Babia Góra NP, Pieniny NP, Wigry NP and Stołowe Góry NP), they were the only invasive species found (Fig. 3; Table S2). A large share (20 species; 29.40%) also had plants from the lowest, I invasiveness category, and the largest number of such species was observed in Wielkopolska NP (15 species; 22.10%), Kampinos NP (12; 17.60%), Słowin NP (12; 17.6%) and Narew NP (11; 16.20%; Fig. 3). Fifteen species belonged to category II (22.10%), and the smallest group (9 species; 13.20%) comprised species classified into III category of invasiveness (Table S2).

**Figure 4:**
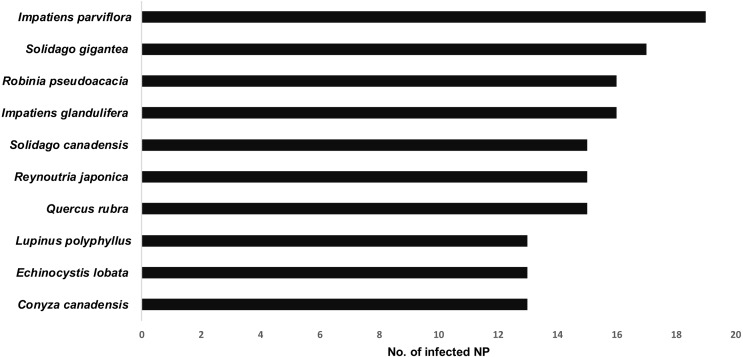
List of the most common of invasive vascular plant species within Polish NPs.

Thirteen species achieved frequency (Fi) above 50%, i.e., occurred in 12 or more national parks (Fig. 4, Table S1). In this group, there were nine species belonging to IV invasive category, including those with the highest frequency: *Impatiens parviflora* (Fi = 82.61%), *Solidago gigantea* (Fi = 73.91%), *Impatiens glandulifera* (Fi = 73.91%), *Reynoutria japonica* (Fi = 73.91%), *Robinia pseudoacacia* (Fi = 69.57%), *Quercus rubra* (Fi = 65.22%), and *Solidago canadensis* (Fi = 65.22%).

Fifteen species occurred only in one or two parks (Fi < 10%; Table S1). Most of them represented I or II category of invasiveness, e.g.: *Alopecurus myosuroides*, *Lysimachia punctata*, *Oxalis corniculata* (each Fi = 4.34%), and *Aronia* x *prunifolia*, *Diplotaxis muralis* (each Fi = 8.69%).

Investigated national parks differed in the number of invasive species, and there were from 2 to 42 taxa in individual parks (Fig. 1, Table 1). The largest number of invasive plants was noted in Drawa NP (42 species; 4.65% of entire vascular flora), Kampinos NP (41; 2.86%), Ojców NP (40; 3.90%), Wielkopolska NP (37; 3.30%), Biebrza NP (33; 3.67%), and Słowin NP (33; 3.62%). Fewer than 10 invasive species were found in five objects: Babia Góra NP (2 species; 0.31% of the entire vascular flora), Karkonosze NP (8; 0.80%), Pieniny NP (7; 0.64%), Stołowe Góry NP (5; 0.77%), and Ujście Warty NP (8; 1.60%). National parks in mountainous areas were usually characterised by a smaller average number of species of invasive plants (min. 2, max. 40, mean 14.8 taxa) than those located in lowland areas (min. 8, max. 42, mean 25.6 taxa).

**Figure 5:**
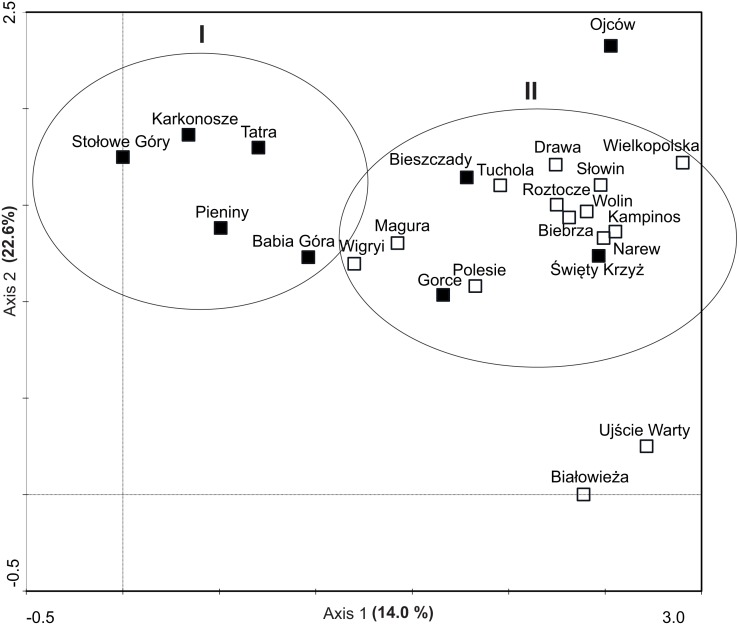
Ordination diagrams of DCA (Detrented Correspondence Analysis) of National Parks based on occurrence invasive alien plants. Explanation: black squares, mountain NP; white squares, lowland NP.

**Figure 6:**
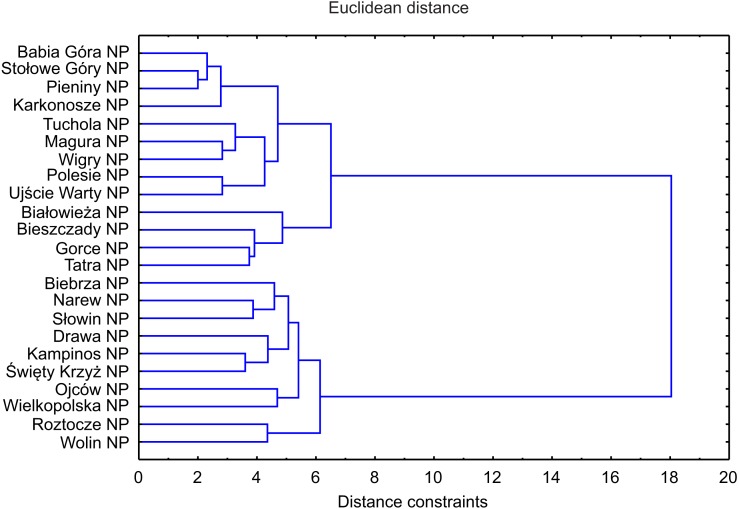
Dendrogram of similarities of invasive plant species occurrence** in NP in Poland** based on Distance constraints.

The DCA led to the identification of two clusters of objects: the first one was composed of five montane national parks with the lowest number of invasive plants, the other one comprised the majority of parks, mainly lowland ones (Fig. 5). The similarity (cluster) analysis conducted showed that lowland objects were floristically similar to each other and formed one, relatively homogeneous, cluster (Fig. 6). The rest of national parks formed the other, more diverse one with clearly separated two subgroups of montane parks. The first one included Babia Góra NP, Karkonosze NP, Pieniny NP and Stołowe Góry NP, while the other one included Bieszczady NP, Gorce NP and Tatra NP.

The number of invasive species decreased with the higher altitude (asl) of the national park (moderate negative correlation, *r* =  − 0.43, *p* < 0.05). By contrast, the number of invasive plants had a positive relationship with the total number of vascular plants, respectively: moderate for lowland NPs (*r* = 0.53, *p* < 0.05), and weak for montane NPs (*r* = 0.35, *p* < 0.05). Moreover, for lowland NPs, the moderate correlation (*r* = 0.45, *p* < 0.05) between the total number of vascular plants and the area of the object was found.

Our results show that 16 invasive alien plant species in total are being managed and that action plans restricting the expansion of invasive plants are currently in place in 12 NPs (Table 1, Table S2)**.** The most frequently eradicated species are *Impatiens glandulifera* (7 out of a total of 17 NPs in which the species occurred), *I. parviflora* (6 out of a 19), *Padus serotina* (4 out a 10) and *Quercus rubra* (4 out a 15). In most parks, management activities are limited to only one species and are most often focused on herbaceous plants, such as *Impatiens glandulifera*, *I. parviflora*, *Heracleum mantegazzianum*, *H. sosnowskyi*, *Reynoutria japonica*, *R. sachalinensis* and *Lupinus polyphyllus*. In a few cases, the management plan includes introduced invasive tree species such as *Padus serotina* and *Quercus rubra* (see the Table S2). Only in five NPs, management actions have been focused on several species, i.e.: Białowieża NP (*Impatiens parviflora*, *Parthenocissus inserta*, *Quercus rubra*), Biebrza NP (*Cornus sericea*, *Echinocystis lobata*, *Lupinus polyphyllus*, *Padus serotina*), Kampinos NP (*Impatiens glandulifera*, *Padus serotina*, *Quercus rubra*, *Reynoutria japonica*), Tuchola NP (*Echinocystis lobata*, *Impatiens glandulifera*, *I. parviflora*, *Quercus rubra*, *Rosa rugosa*), and Wigry NP (*Heracleum sosnowskyi*, *Impatiens glandulifera*, *I. parviflora*).

## Discussion

Our study indicates that even protected areas studied are increasingly affected by invasions of alien plant species. Results of our studies show that the most invasive and most frequent species in Polish NPs (e.g*.*: *Impatiens parviflora*, *Lupinus polyphyllus*, *Padus serotina*, *Quercus rubra*, *Reynoutria japonica*, *Robinia pseudoacacia, Solidago canadensis*, *S. gigantea*) are the same as the most invasive ones on a country scale (Tokarska-Guzik et al., 2012), but also as very invasive ones in Europe, including protected areas (Monaco & Genovesi, 2014; Lapin et al., 2019). These species are troublesome, from an environmental and conservation point of view, as they can compete and even exclude native species, as well as generate economic losses (Rumlerová et al., 2016; Nentwig et al., 2018; Wagner et al., 2017).

Numbers of invasive species in particular national parks varied to a large degree and these differences may have many causes. The number of alien species in PAs is the result of many factors: biotic (e.g.: native species richness, the number of rare species, habitat diversity; Stohlgren et al., 2001; Pyšek, Jarošik & Kučera, 2002; Foxcroft, Rouget & Richardson, 2007; Allen, Brown & Stohlgren, 2009), environmental (climate, topography, hydrography, landscape heterogeneity; McKinney, 2002; Beaumont et al., 2009; Pyšek et al., 2013; Dimitrakopoulos et al., 2017) and anthropogenic (history of anthropogenic land use, human population density, road network, and the number of visitors; Pyšek, Jarošik & Kučera, 2003; Pauchard & Alaback, 2004; Spear et al., 2013).

The conducted research has shown that montane and foothill national parks are more resistant to the penetration of foreign species geographically than parks in the lowlands. This is due to the location of these parks at high altitude, as well as the high naturalness of their protected ecosystems, which limits the penetration of invasive species. This is coincident with the results of Najberek & Solarz (2011), similar dependence was observed also by Pauchard & Alaback (2004) and Kueffer et al. (2013) in the mountains of South America.

On the other hand, national parks where the highest number of invasive species were observed have several common features: they are big (e.g.: Biebrza NP, Kampinos NP), recently established (e.g.: Biebrza NP, Drawa NP, Narew NP, Roztocze NP), have rich flora (e.g.: Wigry NP, Kampinos NP), and finally, are under strong anthropopression (e.g.: Kampinos NP, Ojców NP, Wielkopolska NP). These results support previous findings for protected areas: Fridley, Brown & Bruno (2004) and Allen, Brown & Stohlgren (2009) reported that larger parks have a greater area and therefore more species, regardless whether native or non-native. In turn, Stohlgren et al. (2001) and Stohlgren et al. (2002)) demonstrated an increase in non-native plant species richness with increased native plant richness. As other studies show, the earlier establishment of national park leads to smaller proportions of aliens in flora (Pyšek, Jarošik & Kučera, 2003). Moreover, very strong “infestation” with invasive species of Biebrza NP, Drawa NP, Narew NP and Wigry NP is additionally due to their dominance in the area of wetland ecosystems and the presence of large rivers, particularly strongly exposed to biological invasions (Stohlgren et al., 2001; Stohlgren et al., 2002; Dajdok & Pawlaczyk, 2009). Wetlands, riparian areas and particularly rivers affect invasions by providing areas of high resource availability (Stohlgren et al., 2001; Stohlgren, Loope & Makarick, 2013), periodic disturbances, and a continuous movement of non-native plant species and their propagules downstream (Foxcroft, Rouget & Richardson, 2007).

The history of anthropogenic land use may also favour plant invasions in protected areas (McKinney, 2002; Pyšek, Jarošik & Kučera, 2002; Pyšek et al., 2013), even if disturbances are currently minimised. In Poland, like in many parts of Europe, the majority of national parks have been established in areas which were previously subjected to long anthropogenic pressure (e.g.: Adamowski, Dvorak & Ramanjuk, 2002; Otręba, 2008; Gazda & Szwagrzyk, 2016). Many NPs in Poland were established by the conversion of a forest district (or parts of several forest districts) into a protected area. Thus, alien woody species in Polish NPs are in most cases remnants of former forestry, but also some deliberate introductions already present in the protected areas (Gazda & Szwagrzyk, 2016). The most commonly introduced tree species into the forests (even in the area of NPs) were *Quercus rubra, Padus serotina* and *Robinia pseudoacacia* (Gazda & Szwagrzyk, 2016) along with *Cornus sericea* in Biebrza NP (Werpachowski & Biereżnoj-Bazille, 2015). Although the planting of introduced tree species is currently not allowed in Polish NPs, there is no tendency to eliminate them from managed stands; some of these species are already regenerating naturally and spreading (Danielewicz, 1993; Barabasz-Krasny, Sołtys & Popek, 2004; Otręba & Ferchmin, 2007). Even in the absence of *de novo* introductions of alien species into protected areas, the on-going spread of established (“old”) alien species may severely threaten the conservation value of national parks (Foxcroft et al., 2011; Pyšek et al., 2013).

Introduced tree species are also associated with human settlements; since villages are located within borders of some NPs in Poland (e.g.: Kampinos NP, Wielkopolska NP, Ojców NP), urban settlements may be considered as seed sources of introduced tree species (Otręba & Ferchmin, 2007; Purcel, 2009; Sołtys-Lelek & Barabasz-Krasny, 2010).

Rural areas and forest settlements within the area of national parks are also a source of the dispersal of many invasive plant species (Adamowski, Dvorak & Ramanjuk, 2002; McKinney, 2002; Spear et al., 2013). Numerous ornamental herbaceous plants, climbers, shrubs and trees of alien origin, which not only have settled in but in many cases show territorial expansion, e.g.: *Aster novi-belgii*, *Cornus sericea*, *Helianthus tuberosus*, *Lupinus polyphyllus*, *Reynoutria japonica*, *Rhus typhina*, *Rudbeckia laciniata*, *Solidago canadensis*, *S. gigantea* (Sołtys-Lelek & Barabasz-Krasny, 2010; Kirpluk, 2012; Tałałaj, Brzosko & Pirożnikow, 2013) have been introduced into home gardens located in villages inside and surrounding national parks.

A number of invasive species occurred and spread within national park ecosystems spontaneously, thanks to various factors favouring their dispersal. According to many studies (e.g.: Harrison, Hohn & Ratay, 2002; Gelbard & Belnap, 2003; Pauchard & Alaback, 2004; Allen, Brown & Stohlgren, 2009; Kudo et al., 2014), roads and trailheads are a significant factor in this respect, as they are disturbed and provide pathways for propagule dispersal. Road network and heavy trail use creates disturbed areas, thus boosting available light and other resources for invasive alien plants (Harrison, Hohn & Ratay, 2002). Our results, along with those aforementioned, show that national parks surrounded by a developed network of roads, with numerous trails and routes inside, are vulnerable to invasion, e.g.: Kampinos NP, Ojców NP, Wielkopolska NP (Danielewicz & Maliński, 1997; Sołtys-Lelek & Barabasz-Krasny, 2010; Bomanowska et al., 2014).

Humans are an important vector in the invasion process when they visit national parks. This is visible particularly well in the case of the smallest Polish national park, Ojców NP, which despite rich and unique flora and presence of many precious taxa is heavily invaded, which results from excessive tourist pressure (Sołtys-Lelek & Barabasz-Krasny, 2010). The positive correlation of national park visitation with non-native plant invasions was consistent with the findings of Usher (1988) from reserves in South Africa and North America. However, it is difficult to measure how visitation affects non-native plant richness, whether it is by the direct effect of accidental or intentional propagule introduction into the park or the indirect effect of disturbance (Mack & Lonsdale, 2001) by road development and maintenance (Gelbard & Belnap, 2003) and facilities construction, or many other possible factors.

Summarising, the share of invasive plants in the flora of Polish national parks is far from the maximum values given for protected areas in Europe (40%, Pyšek et al., 2013), and constitutes a maximum of less than 5% of their total flora, which, however, should not be a reason for complacency because invasions are a dynamic process, and the negative effects of the emergence of alien species in the flora may reveal themselves after some time in ways difficult to readily observe (Barney et al., 2013). Several observations suggest that some currently sparse and innocuous species could become invasive. It is an effect of the lag time, i.e., a delay of decades to centuries between the introduction of a species and exponential population growth (Theoharides & Dukes, 2007). Plant invasions can also impact native biota by inducing genetic change in native species. For example, hybridisation between native and non-native plant species is fraught with the risk of losing locally adapted genotypes, and can also lead to fertile hybrids that can displace native species (Yakandawala & Yakandawala, 2011; Daehler & Carino, 2001; Bleeker, Schmitz & Ristow, 2007; Pliszko & Zalewska-Gałosz, 2016).

Taking into consideration negative consequences of the spread of invasive alien plants in protected areas, removing the most obviously invasive species, as well as controlling and prevention of their spread are necessary. Successful management of invasive alien species involves education, prevention, detection and early warning, eradication, containment, and other forms of intervention (Pyšek & Richardson, 2010; Schmiedel et al., 2016). The chances of success of actions undertaken when a species starts to spread in a given area are greater, because the elimination of a species spread over a large area and reaching high numbers in some places is expensive and long-lasting, and sometimes completely impossible (Pyšek & Richardson, 2010). Then only control of existing populations and limiting their numbers remains. Therefore, minimising the risk of the emergence of further species by introducing legal regulations enabling comprehensive activities is equally important as combating the species already present (Pyšek et al., 2013; Genovesi et al., 2015; Foxcroft et al., 2017).

Polish NPs are required to have a management plan in place that is checked and approved by the Ministry of Environment. All management approaches are described in such a management plan which should also include the eradication of invasive species.

The most commonly used methods to manage invasive species in NPs include the manual tearing of individuals or mowing unwanted newcomers and burning the area occupied by them, manual removal of seedlings, cutting saplings and felling bigger trees, and in some cases (e.g.: black cherry), a combination of cutting and spraying with herbicides (Adamowski & Keczyński, 1998; Krzysztofiak & Krzysztofiak, 2015; Gazda & Szwagrzyk, 2016; Obidziński, Kołaczkowska & Otręba, 2016). Unfortunately, these are usually incidental treatments, which significantly reduces their effectiveness. Discontinuity of these management approaches may cause some problems, e.g.: regeneration of the population at the place where the control was carried out, and sometimes the continuation of the species’ spread to new positions (e.g.: Obidziński, Kołaczkowska & Otręba, 2016). Moreover, there are still no available data on the effectiveness of management approaches against invasive plants species in Polish NPs (Gazda & Szwagrzyk, 2016; Obidziński, Kołaczkowska & Otręba, 2016). Summarising, efforts directed towards the elimination of invasive alien plant species from Polish NPs are still in the experimental phase, although the high protection regime, good administrative services and knowledge of local nature support this type of activities in national parks, which ensures higher efficiency of undertaken actions (Obidziński, Kołaczkowska & Otręba, 2016). In addition, legal regulations should prohibit the planting of foreign invasive plant species in national parks and impose significant restrictions in their protection zones (Tałałaj, Brzosko & Pirożnikow, 2013; Obidziński, Kołaczkowska & Otręba, 2016).

Without adequate comprehensive financial and legal strategies, it is impossible to effectively protect areas of high natural value against invasions of alien species (Pyšek et al., 2013; Braun, Schindler & Essl, 2016). This is a difficult task, but as the example of IAS management in Germany (Schmiedel et al., 2016) shows, it can bring effective solutions*.*

## Conclusions

Our research shows that plant invasions occur in all studied protected areas. In total, almost 90% of all invasive plants in Poland were identified in the study. The most widely distributed species with well-documented negative ecological impacts included: *Impatiens parviflora*, *I. glandulifera*, *Solidago gigantea*, *Reynoutria japonica* and *Robinia pseudoacacia*, which is in line with other investigations into the spread of IAS in Europe.

Invasive plants were present in all NPs but analysed objects differ with respect to the number of IAS species and floristic composition of this group. NPs located in lowland areas, with a large surface and subject to strong human pressure, were floristically similar to each other and were characterised by a high number of species of invasive plants. The findings of the study show that elevation is the main driver for the limitation of spread of IAS in protected ecosystems.

The results of the study also show that management of invasive plant species in Polish NPs is often inadequate to substantially reduce the spread of invasive plants in NPs, as there is a lack of comprehensive action plans in the majority of national parks. In most objects, management activities are limited to only one species and have an incidental character. Due to the fact that the problem of invasions does not end at the border of the national park but also (or perhaps above all) affects adjacent areas, it is important to take into account in the management of natural resources local and regional determinants of the occurrence of invasive species.

##  Supplemental Information

10.7717/peerj.8034/supp-1 Table S1 List of invasive vascular plant species occurring in Polish national parks with their characteristics Explanations: T –tree, S –shrub, C –climber, P –perennial, B –biennial, A –annual, Aq –aquatic plant; AR –archaeophyte, KE –kenophyte, ? –dubious status, requires further research; CI –category of invasiveness (according to (Tokarska-Guzik et al., 2012)): I –segetal or ruderal weeds, able to appear in large numbers, mainly on anthropogenic habitats, or potentially invasive species, currently occupying limited acreage or having a small number of localities in the country or in individual regions, II –species in which invasive properties are already detected in some regions from increasing area of occupancy or number of localities, or which are characterized by previously observed invasive behaviour in other countries, III –species which occur in a few localities in large numbers or are scattered over many localities, admittedly in small numbers but with known negative impact on native species, habitats and ecosystems and/or on the economy and society, IV –the most dangerous invasive plants, the significance of the presence of those species in Poland is fundamental –both a substantial amount of localities, and large local populations are known; most are still increasing in number of localities or area of occupancy. The species from IV category are in grey. Species listed in the Regulation of the Polish Minister of the Environment of November 11th 2011 are in bold. These species require permission and must meet certain standards for being imported, kept, cultivated/bred or traded; Fi –frequency.

10.7717/peerj.8034/supp-2 Table S2 List of invasive vascular plant species occurring in Polish national parks * species eradicated in NPs, in brackets number of NPs where the species are managed, ’1’: the presence of a species in NPs, missing value: the absence of a species in NPs

## Additional Information and Declarations

### Data Availability

The following information was supplied regarding data availability: The raw data is available in the Supplemental Tables.
