# Supplementary material for: Invasive alien plants in Polish national parks—threats to species diversity
Source: PeerJ. 2019 Dec 13;7:e8034. doi: 10.7717/peerj.8034 (PMC6913259; doi:10.7717/peerj.8034)
Supplement: Table S1 — Explanations: T –tree, S –shrub, C –climber, P –perennial, B –biennial, A –annual, Aq –aquatic plant; AR –archaeophyte, KE –kenophyte, ? –dubious status, requires further research; CI –category of invasiveness (according to (Tokarska-Guzik et al., 2012)): I –segetal or ruderal weeds, able to appear in large numbers, mainly on anthropogenic habitats, or potentially invasive species, currently occupying limited acreage or having a small number of localities in the country or in individual regions, II –species in which invasive properties are already detected in some regions from increasing area of occupancy or number of localities, or which are characterized by previously observed invasive behaviour in other countries, III –species which occur in a few localities in large numbers or are scattered over many localities, admittedly in small numbers but with known negative impact on native species, habitats and ecosystems and/or on the economy and society, IV –the most dangerous invasive plants, the significance of the presence of those species in Poland is fundamental –both a substantial amount of localities, and large local populations are known; most are still increasing in number of localities or area of occupancy. The species from IV category are in grey. Species listed in the Regulation of the Polish Minister of the Environment of November 11th 2011 are in bold. These species require permission and must meet certain standards for being imported, kept, cultivated/bred or traded; Fi –frequency. [file peerj-07-8034-s001.docx]

| No. | Species | Family | Life span | Origin – native range | Geographical-historical group | CI | No. of infected NP | Fi [%] |
| --- | --- | --- | --- | --- | --- | --- | --- | --- |
| 1 | *Acer negundo* L. | Aceraceae | T | N Am | KE | IV | 12 | 52.17 |
| 2 | ***Ailanthus altissima*** (Mill.) Swingle | Simaroubaceae | T | E Asia [China] | KE | III | 1 | 4.34 |
| 3 | *Alopecurus myosuroides* Huds. | Poaceae | A | Medit, SW Asia | AR | I | 1 | 4.34 |
| 4 | *Amaranthus retroflexus* L. | Amaranthaceae | A | N&C Am | KE | I | 8 | 34.78 |
| 5 | *Ambrosia artemisiifolia* L. | Asteraceae | A | N Am | KE | IV | 1 | 4.34 |
| 6 | *Amelanchier lamarckii* F.G. Schroed | Rosaceae | T/S | N Am | KE | II | 1 | 4.34 |
| 7 | *Amelanchier spicata* (Lam.) K.Koch | Rosaceae | S | N Am | KE | IV | 4 | 17.39 |
| 8 | *Anthoxanthum aristatum* Boiss. | Poaceae | A | S Europe | KE | I | 3 | 13.04 |
| 9 | *Aronia x prunifolia* (Marshall) Rehder | Rosaceae | S | N Am | KE | II | 2 | 8.69 |
| 10 | *Aster novi-belgii* L. | Asteraceae | P | N Am | KE | IV | 6 | 26.08 |
| 11 | *Aster ×salignus* Willd. | Asteraceae | P | N Am | KE | I | 2 | 8.69 |
| 12 | *Avena fatua* L. s.l. | Poaceae | A | SW Asia | AR | I | 7 | 30.43 |
| 13 | *Bidens frondosa* L. | Asteraceae | A | N Am | KE | III | 10 | 43.47 |
| 14 | *Bromus carinatus* Hook. & Arn. | Poaceae | A | N Am | KE | IV | 6 | 26.08 |
| 15 | *Bunias orientalis* L. | Brassicaceae | A | SE Europe, W Asia | KE? | III | 7 | 30.43 |
| 16 | *Clematis vitalba* L. | Ranunculaceae | P | S&W&C Europe, NW Africa, Asia [Caucasus] | KE | II | 4 | 17.39 |
| 17 | *Conyza canadensis* (L.) Cronquist | Asteraceae | C | N Am | KE | I | 13 | 56.52 |
| 18 | *Cornus sericea* L.emend. Murray | Cornaceae | A | N Am | KE | III | 5 | 21.73 |
| 19 | *Digitalis purpurea* L. | Poaceae | P | W Europe | KE | II | 5 | 21.73 |
| 20 | *Diplotaxis muralis* (L.) DC. | Brassicaceae | A | S&W Europe, [Africa] | KE | I | 2 | 8.69 |
| 21 | *Echinochloa crus-galli* (L.) P.Beauv | Poaceae | A | Asia | AR | I | 9 | 39.13 |
| 22 | ***Echinocystis lobata*** **(F.Michx) Torr. & A. Gray** | Cucurbitaceae | A | N Am | KE | IV | 13 | 56.52 |
| 23 | *Elodea canadensis* Michx. | Hydrocharitaceae | P/Aq | N Am | KE | IV | 11 | 47.82 |
| 24 | *Epilobium ciliatum* Raf. | Onagraceae | P | N Am | KE | II | 6 | 26.08 |
| 25 | *Erigeron annuus* (L.) Pers | Asteraceae | P | N Am | KE | II | 10 | 43.47 |
| 26 | *Fraxinus pennsylvanica* Marshall | Oleaceae | T | N Am | KE | III | 5 | 21.73 |
| 27 | *Galinsoga ciliata* (Raf.) S.F. Blake | Asteraceae | A | C&S Am | KE | I | 11 | 47.82 |
| 28 | *Galinsoga parviflora* Cav. | Asteraceae | A | C&S Am | KE | I | 10 | 43.47 |
| 29 | *Helianthus tuberosus* L. | Asteraceae | P | N Am | KE | II | 12 | 51.17 |
| 30 | ***Heracleum mantegazzianum* Sommier & Levier** | Apiaceae | B/P | Caucasus | KE | IV | 5 | 21.73 |
| 31 | ***Heracleum sosnowskyi* Manden.** | Apiaceae | P | Caucasus | KE | IV | 8 | 34.78 |
| 32 | *Hordeum murinum* L. | Poaceae | A | Europe, Asia | AR | I | 3 | 13.04 |
| 33 | ***Impatiens capensis* Meerb.** | Balsaminaceae | A | N Am | KE | III | 1 | 4.34 |
| 34 | ***Impatiens glandulifera* Royle** | Balsaminaceae | A | Himalaya | KE | IV | 16 | 69.56 |
| 35 | *Impatiens parviflora* DC. | Balsaminaceae | A | C&E Asia | KE | IV | 19 | 82.6 |
| 36 | *Juglans regia* L. | Juglandaceae | T | SW&C&E Asia | KE | II | 5 | 21.73 |
| 37 | *Juncus tenuis* Willd. | Juncaceae | P | N Am | KE | I | 12 | 51.17 |
| 38 | *Lemna turionifera* Landolt | Lemnaceae | P/Aq | N Am | KE | III | 1 | 4.34 |
| 39 | *Lolium multiflorum* Lam. | Poaceae | P/A | S&W Europe, SW Asia, N Africa | KE | II | 4 | 17.39 |
| 40 | *Lupinus polyphyllus* Lindl. | Fabaceae | P | N Am | KE | III | 13 | 56.52 |
| 41 | *Lycium barbarum* L. | Solanaceae | S | SE Europe, E Asia | KE | I | 6 | 26.08 |
| 42 | *Lysimachia punctata* L. | Primulaceae | P | SE Europe | KE | I | 1 | 4.34 |
| 43 | *Mimulus guttatus* DC. | Scrophulariaceae | P | N Am | KE | III | 3 | 13.04 |
| 44 | *Onobrychis viciifolia* .Scop. | Fabaceae | P | S&SE Europe | KE | II | 1 | 4.34 |
| 45 | *Oxalis corniculata* L. | Oxalidaceae | A/B/P | S Europe, SW Asia, Africa, Australia | KE | I | 1 | 4.34 |
| 46 | *Oxalis fontana* Bunge | Oxalidaceae | A/B/P | N Am | KE | I | 7 | 30.43 |
| 47 | *Padus serotina* (Ehrh.) Borkh. | Rosaceae | T | N&C Am | KE | IV | 10 | 43.47 |
| 48 | *Parthenocissus inserta* (A. Kern.) Fritsch | Vitaceae | C | N Am | KE | II | 6 | 26.08 |
| 49 | *Quercus rubra* L. | Fagaceae | T | N Am | KE | IV | 15 | 60.86 |
| 50 | ***Reynoutria* x*bohemica* Chrtek et Chrtková** | Polygonaceae | P | Anthropog | KE | IV | 2 | 8.69 |
| 51 | ***Reynoutria japonica* (Houtt.) Ronse Decraene** | Polygonaceae | P | E Asia | KE | IV | 15 | 65.21 |
| 52 | ***Reynoutria sachalinensis* (F. Schmidt) Nakai** | Polygonaceae | P | E Asia | KE | IV | 7 | 30.43 |
| 53 | *Rhus typhina* L. | Anacardiaceae | T/S | N Am | KE | II | 6 | 26.08 |
| 54 | *Robinia pseudoacacia* L. | Fabaceae | T | N Am | KE | IV | 16 | 69.56 |
| 55 | *Rosa rugosa* Thunb. | Rosaceae | S | E Asia | KE | IV | 11 | 47.82 |
| 56 | *Rudbeckia laciniata* L. | Asteraceae | P | N Am | KE | IV | 11 | 47.82 |
| 57 | *Rumex confertus* Willd. | Polygonaceae | P | SE Europe, W Asia | KE | II | 3 | 13.04 |
| 58 | *Setaria pumila* (Poir.) Roem.et Schult. | Poaceae | A | S&SE Asia | AR | I | 7 | 30.43 |
| 59 | *Setaria viridis* (L.) Beauv. | Poaceae | A | Medit., SW Asia | AR | I | 7 | 30.43 |
| 60 | *Solidago canadensis* L. | Asteraceae | P | N Am | KE | IV | 15 | 65.21 |
| 61 | *Solidago gigantea* Aiton | Asteraceae | P | N Am | KE | IV | 17 | 73.91 |
| 62 | *Solidago graminifolia* (L.) Elliott | Asteraceae | P | N Am | KE | IV | 2 | 8.69 |
| 63 | *Spiraea tomentosa* L. | Rosaceae | S | N Am | KE | IV | 1 | 4.34 |
| 64 | *Telekia speciosa* (Schreb.) Baumg. | Asteraceae | P | Europe | KE? | II | 3 | 13.04 |
| 65 | *Veronica filiformis* Sm. | Scrophulariaceae | P | Caucasus | KE | II | 3 | 13.04 |
| 66 | *Veronica persica* Poir. | Scrophulariaceae | A | Caucasus | KE | I | 11 | 47.82 |
| 67 | *Vicia grandiflora* Scop. | Fabaceae | A | S Europe, SW Asia | KE | I | 1 | 4.34 |
| 68 | *Xanthium albinum* (Widder) H. Scholz | Asteraceae | A | N Am | KE | IV | 3 | 13.04 |
