# Supplementary material for: Invasive alien plants in Polish national parks—threats to species diversity
Source: PeerJ. 2019 Dec 13;7:e8034. doi: 10.7717/peerj.8034 (PMC6913259; doi:10.7717/peerj.8034)
Supplement: Table S2 — * species eradicated in NPs, in brackets number of NPs where the species are managed, ’1’: the presence of a species in NPs, missing value: the absence of a species in NPs [file peerj-07-8034-s002.docx]

| **Nazwa łacińska** | Babia Góra NP | Białowieża NP | Biebrza NP | Bieszczady NP | Drawa NP | Gorce NP | Kampinos NP | Karkonosze NP | Magura NP | Narew NP | Ojców NP | Pieniny NP | Polesie NP | Roztocze NP | Słowin NP | Stołowe Góry. NP | Święty Krzyż NP | Tatra NP | Tuchola NP | Ujście Warty NP | Wielkopolska NP | Wigry NP | Wolin NP | TOTAL | Species invasive in European PAs (Monaco, Genovesi 2014) |
| --- | --- | --- | --- | --- | --- | --- | --- | --- | --- | --- | --- | --- | --- | --- | --- | --- | --- | --- | --- | --- | --- | --- | --- | --- | --- |
| *Acer negundo* L. |  | 1 | 1* |  | 1 |  | 1 |  |  | 1 |  |  | 1 | 1 | 1 |  | 1 |  |  | 1 | 1 | 1* |  | 12 (2) | + |
| *Ailanthus altissima* (Mill.) Swingle |  |  |  |  |  |  |  |  |  |  |  |  |  |  |  |  |  |  |  |  | 1 |  |  | 1 | + |
| *Alopecurus myosuroides* Huds. |  |  |  |  |  |  |  |  |  |  | 1 |  |  |  |  |  |  |  |  |  |  |  |  | 1 |  |
| *Amaranthus retroflexus* L. |  |  | 1 |  | 1 |  | 1 |  |  | 1 | 1 |  |  |  | 1 |  | 1 |  |  |  | 1 |  |  | 8 |  |
| *Ambrosia artemisiifolia* L. |  |  |  |  |  |  |  |  |  |  | 1 |  |  |  |  |  |  |  |  |  |  |  |  | 1 | + |
| *Amelanchier lamarckii* F.G. Schroed |  | 1 |  |  |  |  |  |  |  |  |  |  |  |  |  |  |  |  |  |  |  |  |  | 1 |  |
| *Amelanchier spicata* (Lam.) K.Koch |  |  |  |  | 1 |  | 1 |  |  |  |  |  |  | 1 |  |  |  |  |  |  | 1 |  |  | 4 |  |
| *Anthoxanthum aristatum* Boiss. |  |  |  |  |  |  | 1 |  |  |  |  |  |  |  | 1 |  |  |  |  |  | 1 |  |  | 3 |  |
| *Aronia x prunifolia* (Marshall) Rehder |  |  |  |  |  |  |  |  |  |  |  |  |  | 1 |  |  |  |  |  |  |  |  | 1 | 2 |  |
| *Aster novi-belgii* L. |  |  |  |  | 1 |  | 1 |  |  |  | 1 |  |  |  | 1 |  | 1 |  |  |  | 1 |  |  | 6 |  |
| *Aster ×salignus* Willd. |  |  | 1 |  |  |  |  |  |  |  | 1 |  |  |  |  |  |  |  |  |  |  |  |  | 2 |  |
| *Avena fatua* L. s.l. |  |  |  |  | 1 |  | 1 |  |  | 1 | 1 |  |  | 1 | 1 |  |  |  |  |  | 1 |  |  | 7 |  |
| *Bidens frondosa* L. |  | 1 | 1 |  | 1 | 1 | 1 |  |  | 1 |  |  |  |  |  |  | 1 |  |  | 1 | 1 |  | 1 | 10 | + |
| *Bromus carinatus* Hook. & Arn. |  |  | 1 |  | 1 |  | 1 |  |  |  | 1 |  |  |  | 1 |  |  |  |  |  | 1 |  |  | 6 |  |
| *Bunias orientalis* L. |  |  | 1 | 1 |  | 1 |  |  |  | 1 | 1 |  |  |  |  |  |  | 1 |  |  | 1 |  |  | 7 |  |
| *Clematis vitalba* L. |  |  |  |  | 1 |  |  |  |  |  | 1 |  |  |  | 1 |  |  |  |  |  | 1 |  |  | 4 |  |
| *Conyza canadensis* (L.) Cronquist |  | 1 | 1 | 1 | 1 | 1 | 1 |  |  | 1 | 1 |  | 1 |  | 1 |  | 1 |  |  | 1 | 1 |  | 1 | 14 |  |
| *Cornus sericea* L.emend. Murray |  |  | 1* |  |  | 1 | 1 |  |  | 1 |  |  |  |  | 1 |  |  |  |  |  | 1 |  |  | 6 (1) | + |
| *Digitalis purpurea* L. |  |  |  |  | 1 |  |  |  |  |  |  |  |  | 1 | 1 |  |  | 1 |  |  |  |  | 1 | 5 |  |
| *Diplotaxis muralis* (L.) DC. |  |  |  |  |  |  |  |  |  |  | 1 |  |  |  |  |  |  |  |  |  | 1 |  |  | 2 |  |
| *Echinochloa crus-galli* (L.) P.Beauv |  |  | 1 |  | 1 |  | 1 |  |  | 1 | 1 |  |  | 1 | 1 |  | 1 |  |  |  | 1 |  |  | 9 |  |
| *Echinocystis lobata* (F.Michx) Torr. & A. Gray |  | 1 | 1* |  | 1 |  | 1 |  | 1 | 1 | 1 |  |  | 1 | 1 |  | 1 |  | 1* |  |  | 1 | 1 | 13 (2) | + |
| *Elodea canadensis* Michx. |  | 1 | 1 |  | 1 |  | 1 |  |  | 1 |  |  |  | 1 | 1 |  | 1 |  |  | 1 | 1 |  | 1 | 11 | + |
| *Epilobium ciliatum* Raf. |  | 1 |  |  | 1 | 1 | 1 |  |  |  |  |  |  |  |  |  | 1 | 1 |  |  |  |  |  | 6 |  |
| *Erigeron annuus* (L.) Pers |  | 1 | 1 | 1 | 1 | 1 | 1 |  |  |  | 1 |  |  | 1 |  |  | 1 |  |  |  | 1 |  |  | 10 | + |
| *Fraxinus pennsylvanica* Marshall |  |  |  |  | 1 |  | 1 |  |  |  |  |  |  | 1 |  |  | 1 |  | 1 |  |  |  |  | 5 |  |
| *Galinsoga ciliata* (Raf.) S.F. Blake |  |  | 1 | 1 | 1 | 1 | 1 |  |  | 1 | 1 |  |  | 1 | 1 |  | 1 |  |  |  | 1 |  | 1 | 12 |  |
| *Galinsoga parviflora* Cav. |  |  | 1 |  | 1 |  | 1 |  |  | 1 | 1 |  |  | 1 | 1 |  | 1 |  |  |  | 1 |  | 1 | 10 |  |
| *Helianthus tuberosus* L. |  |  | 1* |  | 1 |  | 1 |  | 1 | 1 | 1 |  | 1 | 1 |  |  | 1 |  | 1 |  | 1 |  | 1 | 12 (1) | + |
| *Heracleum mantegazzianum* Sommier & Levier |  |  | 1 | 1 |  |  | 1 |  |  |  |  |  |  |  |  | 1 |  |  |  |  |  | 1 |  | 5 | + |
| *Heracleum sosnowskyi* Manden. |  |  | 1 |  |  |  |  | 1* | 1 |  |  | 1* |  | 1 |  | 1 |  | 1 |  |  |  | 1* |  | 8 | + |
| *Hordeum murinum* L. |  |  |  |  | 1 |  |  |  |  |  | 1 |  |  |  |  |  |  |  |  |  | 1 |  |  | 3 |  |
| *Impatiens capensis* Meerb. |  |  |  |  |  |  |  |  |  |  |  |  |  |  |  |  |  |  |  |  |  |  | 1 | 1 |  |
| *Impatiens glandulifera* Royle | 1 |  | 1* | 1 | 1 | 1 | 1* | 1* | 1* | 1 | 1 | 1 |  |  | 1 | 1 | 1 | 1* | 1* |  |  | 1* |  | 17 (7) | + |
| *Impatiens parviflora* DC. | 1* | 1* | 1* |  | 1 | 1 | 1 |  | 1 | 1 | 1 | 1 | 1 | 1 | 1 |  | 1 |  | 1* | 1 | 1 | 1* | 1* | 19 (6) | + |
| *Juglans regia* L. |  |  | 1 |  | 1 |  |  |  |  |  | 1 |  |  |  |  |  | 1 |  |  |  |  |  | 1 | 5 |  |
| *Juncus tenuis* Willd. |  | 1 |  | 1 | 1 | 1 | 1 |  |  | 1 | 1 |  |  |  | 1 |  | 1 | 1 |  |  | 1 |  | 1 | 12 |  |
| *Lemna turionifera* Landolt |  |  |  |  |  |  |  |  |  | 1 |  |  |  |  |  |  |  |  |  |  |  |  |  | 1 |  |
| *Lolium multiflorum* Lam. |  |  |  |  |  | 1 |  |  |  | 1 | 1 |  |  |  | 1 |  | 1 |  |  |  |  |  |  | 5 |  |
| *Lupinus polyphyllus* Lindl. |  | 1 | 1* | 1 | 1 | 1 | 1 | 1* |  |  | 1 |  |  | 1 | 1 |  | 1 |  | 1 |  | 1 |  |  | 13 (2) | + |
| *Lycium barbarum* L. |  |  | 1 |  | 1 |  | 1 |  |  |  | 1 |  |  |  | 1 |  |  |  |  |  | 1 |  |  | 6 |  |
| *Lysimachia punctata* L. |  |  |  |  |  |  |  |  |  |  |  |  |  |  |  |  |  | 1 |  |  |  |  |  | 1 |  |
| *Mimulus guttatus* DC. |  |  |  |  | 1 |  |  | 1* |  |  |  |  |  |  | 1 |  |  |  |  |  |  |  |  | 3 (1) |  |
| *Onobrychis viciifolia* .Scop. |  |  |  |  |  |  |  |  |  |  | 1 |  |  |  |  |  |  |  |  |  |  |  |  | 1 |  |
| *Oxalis corniculata* L. |  | 1 |  |  |  |  |  |  |  |  |  |  |  |  |  |  |  |  |  |  |  |  |  | 1 |  |
| *Oxalis fontana* Bunge |  |  |  | 1 |  | 1 | 1 |  |  | 1 | 1 |  |  | 1 |  |  | 1 |  |  |  | 1 |  |  | 8 |  |
| *Padus serotina* (Ehrh.) Borkh. |  |  | 1* |  |  |  | 1* |  |  | 1 |  |  | 1 | 1* | 1 |  | 1 |  | 1 |  |  | 1* | 1 | 10 (4) | + |
| *Parthenocissus inserta* (A. Kern.) Fritsch |  | 1* | 1 |  |  |  | 1 |  |  |  | 1 |  |  | 1 |  |  | 1 |  |  |  |  |  |  | 6 (1) |  |
| *Quercus rubra* L. |  | 1* | 1 |  | 1 |  | 1* |  | 1 | 1 | 1 |  | 1 | 1 | 1 |  | 1 |  | 1* |  | 1 | 1* | 1 | 15 (4) |  |
| *Reynoutria* x*bohemica* Chrtek et Chrtková |  |  |  |  |  |  | 1 |  |  |  | 1 |  |  |  |  |  |  |  |  |  |  |  |  | 2 | + |
| *Reynoutria japonica* (Houtt.) Ronse Decraene |  | 1 | 1 |  | 1 | 1 | 1* |  | 1 |  | 1 | 1 | 1 | 1 | 1 | 1 | 1 | 1 |  |  | 1 | 1* | 1 | 17 (2) | + |
| *Reynoutria sachalinensis* (F. Schmidt) Nakai |  |  |  |  | 1 |  | 1 | 1* |  |  | 1 | 1 | 1 |  |  | 1 |  |  |  |  |  |  |  | 7 (1) | + |
| *Rhus typhina* L. |  |  |  |  | 1 |  | 1 |  |  |  | 1 |  |  | 1 |  |  |  |  | 1 |  |  |  | 1 | 6 |  |
| *Robinia pseudoacacia* L. |  |  | 1* |  | 1 | 1 | 1 |  | 1 | 1 | 1 |  | 1 | 1 | 1 |  | 1 |  | 1 | 1 | 1 | 1* | 1 | 16 (2) | + |
| *Rosa rugosa* Thunb. |  |  | 1 | 1 | 1 | 1 |  |  |  | 1 | 1 |  |  |  | 1 |  |  | 1 | 1* |  | 1 | 1* | 1 | 12 (2) |  |
| *Rudbeckia laciniata* L. |  | 1 | 1 | 1 | 1 |  | 1 |  | 1 |  | 1 |  |  | 1 | 1 |  | 1 |  |  |  | 1 |  |  | 11 |  |
| *Rumex confertus* Willd. |  | 1 | 1 | 1 |  |  |  |  |  |  |  |  |  |  |  |  |  |  |  |  |  |  |  | 3 |  |
| *Setaria pumila* (Poir.) Roem.et Schult. |  | 1 |  |  | 1 |  | 1 |  |  | 1 |  |  |  |  | 1 |  | 1 |  |  |  | 1 |  |  | 7 |  |
| *Setaria viridis* (L.) Beauv. |  | 1 | 1 |  | 1 |  | 1 |  |  | 1 |  |  |  |  | 1 |  | 1 |  |  |  |  |  |  | 7 |  |
| *Solidago canadensis* L. |  |  |  |  | 1 | 1 | 1 | 1 |  | 1 | 1 | 1 | 1 | 1 | 1 |  | 1 |  |  | 1 | 1 | 1* | 1 | 15 (1) | + |
| *Solidago gigantea* Aiton |  | 1 | 1 | 1 | 1 | 1 | 1 | 1 | 1 | 1 | 1 | 1 | 1 | 1 | 1 |  | 1 |  |  |  | 1 | 1 |  | 17 | + |
| *Solidago graminifolia* (L.) Elliott |  |  |  |  | 1 |  |  | 1 |  |  |  |  |  |  |  |  |  |  |  |  |  |  |  | 2 |  |
| *Spiraea tomentosa* L. |  |  |  |  | 1 |  |  |  |  |  |  |  |  |  |  |  |  |  |  |  |  |  |  | 1 |  |
| *Telekia speciosa* (Schreb.) Baumg. |  |  |  | 1 | 1 |  |  |  |  |  |  |  |  |  |  |  |  | 1 |  |  |  |  |  | 3 | + |
| *Veronica filiformis* Sm. |  |  |  | 1 |  | 1 |  |  |  |  | 1 |  |  |  |  |  |  |  |  |  |  |  |  | 3 |  |
| *Veronica persica* Poir. |  |  | 1 | 1 | 1 | 1 | 1 |  |  | 1 | 1 |  |  | 1 | 1 |  | 1 | 1 |  |  | 1 |  |  | 12 |  |
| *Vicia grandiflora* Scop. |  |  |  |  |  |  |  |  |  |  |  |  |  |  |  |  |  |  |  |  | 1 |  |  | 1 |  |
| *Xanthium albinum* (Widder) H. Scholz |  |  |  |  |  |  | 1 |  |  |  |  |  |  |  |  |  |  |  |  | 1 | 1 |  |  | 3 |  |
| TOTAL | 2 | 20 | 33 | 16 | 42 | 20 | 41 | 8 | 10 | 28 | 40 | 7 | 11 | 27 | 33 | 5 | 32 | 11 | 11 | 8 | 37 | 13 | 20 |  |  |
